# Improved analysis of C_4_ and C_3_ photosynthesis via refined *in vitro* assays of their carbon fixation biochemistry

**DOI:** 10.1093/jxb/erw154

**Published:** 2016-04-27

**Authors:** Robert E. Sharwood, Balasaheb V. Sonawane, Oula Ghannoum, Spencer M. Whitney

**Affiliations:** ^1^ARC Centre of Excellence for Translational Photosynthesis, Research School of Biology, Australian National University, Canberra ACT 2601, Australia; ^2^ARC Centre of Excellence for Translational Photosynthesis, Hawkesbury Institute for the Environment, Western Sydney University, Richmond NSW 2753, Australia

**Keywords:** Carbamylation, carbon fixation, CO_2_-concentrating mechanism, photosynthesis, Rubisco, Rubisco activase.

## Abstract

Plants operating C_3_ and C_4_ photosynthetic pathways exhibit differences in leaf anatomy and photosynthetic carbon fixation biochemistry. Fully understanding this underpinning biochemical variation is requisite to identifying solutions for improving photosynthetic efficiency and growth. Here we refine assay methods for accurately measuring the carboxylase and decarboxylase activities in C_3_ and C_4_ plant soluble protein. We show that differences in plant extract preparation and assay conditions are required to measure NADP-malic enzyme and phosphoenolpyruvate carboxylase (pH 8, Mg^2+^, 22 °C) and phosphoenolpyruvate carboxykinase (pH 7, >2mM Mn^2+^, no Mg^2+^) maximal activities accurately. We validate how the omission of MgCl_2_ during leaf protein extraction, lengthy (>1min) centrifugation times, and the use of non-pure ribulose-1,5-bisphosphate (RuBP) significantly underestimate Rubisco activation status. We show how Rubisco activation status varies with leaf ontogeny and is generally lower in mature C_4_ monocot leaves (45–60% activation) relative to C_3_ monocots (55–90% activation). Consistent with their >3-fold lower Rubisco contents, full Rubisco activation in soluble protein from C_4_ leaves (<5min) was faster than in C_3_ plant samples (<10min), with addition of Rubisco activase not required for full activation. We conclude that Rubisco inactivation in illuminated leaves primarily stems from RuBP binding to non-carbamylated enzyme, a state readily reversible by dilution during cellular protein extraction.

## Highlight

We provide improved biochemical assays for key carboxylases and decarboxylases involved in C_3_ and C_4_ photosynthesis. Together with leaf age-related changes in Rubisco activation, these measurements will improve photosynthetic modelling.

## Introduction

Plants operating the C_3_ and C_4_ pathways contain differing biochemical and anatomical features that facilitate their climatic adaptation to cool-temperate and warm-tropical environments, respectively (Edwards *et al.*, 2010). The rate-limiting CO_2_ fixation step common to both pathways is catalysed by the photosynthetic enzyme ribulose 1,5-bisphosphate (RuBP) carboxylase/oxygenase (Rubisco, EC 4.1.1.39). Fixation of CO_2_ to RuBP by Rubisco produces two molecules of 3-phosphoglycerate (PGA) that are cycled through the photosynthetic carbon reduction (PCR) cycle to produce triose-phosphates, the building blocks of carbohydrates needed for plant growth (Raines, 2003).

Rubisco is an imperfect catalyst that is remarkably slow (completing only 2–4 cycles s^–1^ in leaves) and can fix O_2_ instead of CO_2_, with oxygenation of RuBP leading to the production of 2-phosphoglycolate (PGly) whose recycling back to PGA by the photorespiratory pathway spans three cellular compartments and undesirably consumes energy and releases fixed CO_2_ (Bauwe *et al.*, 2010). Overcoming the catalytic limitations of Rubisco by directed changes to the enzyme or concentrating CO_2_ around Rubisco to reduce the costs of photorespiration are ongoing bioengineering challenges (Parry *et al.*, 2013; Price *et al.*, 2013; Long *et al.*, 2015). Current efforts to generate or discover plant Rubisco isoforms with joint improvements in specificity for CO_2_ as opposed to O_2_ (S_c/o_) and carboxylation efficiency (defined as the maximum carboxylation rate (*k*_cat_^c^) divided by the *K*_m_ for CO_2_ under ambient O_2_; *K*_C_^21%O2^) have yet to yield success (Sharwood and Whitney, 2014), despite such Rubiscos existing in some non-green algae (Andrews and Whitney, 2003).

The evolution of C_4_ photosynthesis ~35–40 million years ago provided a natural solution to remedy the inefficiency of Rubisco (Sage *et al.*, 2012). The anatomical separation of phosphoenolpyruvate carboxylase (PEPC) in mesophyll cells (MCs) and Rubisco in the bundle sheath cell (BSC) chloroplasts was accompanied by adaptation of biochemical CO_2_-concentrating mechanisms (CCMs) (Hatch, 1987; Kanai and Edwards, 1999). The C_4_ pathway involves the hydration by carbonic anhydrase of CO_2_ to HCO_3_^−^ which is combined with phosphoenolpyruvate (PEP) by phosphoenolpyruvate carboxylase (PEPC) into the 4C acid oxaloacetate (OAA) that is converted into malate or aspartate before diffusing into the BSCs where they are decarboxylated, raising the CO_2_ around Rubisco >4-fold higher than ambient CO_2_ (von Caemmerer, 2000; Sage, 2004). The 3C decarboxylation product, pyruvate, returns to the MCs for PEP regeneration by pyruvate phosphate dikinase (PPDK) at the cost of two ATP equivalents.

The ratio of the activity between the main carboxylases is a key determinant of the efficiency of the CCM and can be an indication of the CO_2_ supply to BSCs (von Caemmerer *et al.*, 2014). On the one hand, PEPC activity is much higher than that of Rubisco (2- to 10-fold depending on plant species and environmental conditions) to enable the C_4_ acid gradient to build and facilitate the diffusion of the C_4_ acids into the BSCs. On the other hand, the PEPC:Rubisco ratio must be optimized to minimize CO_2_ leakage from the BSCs, leading to futile cycling involving the CCM (Kromdijk *et al.*, 2008). Futile cycling of the CCM is energetically wasteful for the plant through use of ATP to regenerate PEP.

Based on the main decarboxyating enzymes, C_4_ plants can be grouped into three biochemical subtypes: NADP-malic enzyme (NADP-ME), NAD-malic enzyme (NAD-ME), and phosphoenolpyruvate carboxykinase (PEPCK) (Hatch, 1987; Kanai and Edwards, 1999). In addition, there is flexibility among some NADP-ME (e.g maize, sorghum, and *Panicum antidotale*) and NAD-ME (e.g. *Cleome angustifolia*, *Bienertia sinuspersici*, and *Panicum coloratum*) species which also harbour PEPCK (Furbank, 2011; Pinto *et al.*, 2014; Koteyeva *et al.*, 2015). The significance of the dual decarboxylation pathways is not yet fully understood, but evidence suggests that PEPCK allows flexibility to the decarboxylation pathway(s) that may be dependent on environmental cues (Bellasio and Griffiths, 2014; Sharwood *et al.*, 2014). Assaying for PEPCK is often difficult because pure enzyme is required for assaying in the decarboxylase direction due to interference from other C_4_ pathway enzymes (Ashton *et al.*, 1990). However, assaying for PEPCK activity in the carboxylase direction is also troublesome as PEPC can interfere with this assay, although variations in PEPCK activities can be corroborated by western blot analysis of PEPCK content (Sharwood *et al.*, 2014). Accurately determining the level of maximum PEPCK activity is requisite to determine the level of flexibility in the decarboxylation pathways that may exist.

**Fig. 1.**
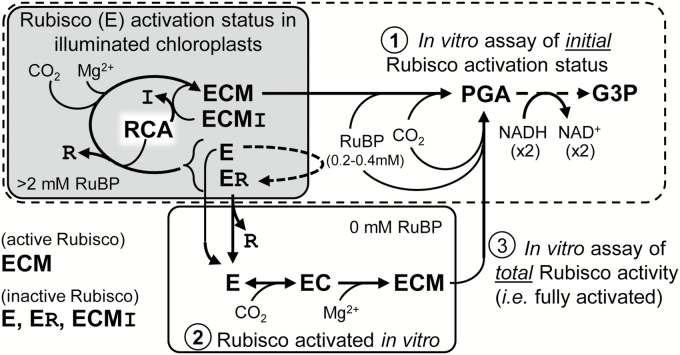
Summary of Rubisco activation status *in vivo* and modulation *in vitro*. Summary of the NADH-linked assay used to determine Rubisco activation status using rapidly extracted soluble protein from young, non-stressed leaves sampled during illumination [where inactive ECMI complexes containing the Rubisco inhibitors (I) CA1P or XuBP would be negligible]. See text for details of the three assay stages indicated. R, RuBP; RCA, Rubisco activase. Details of the NADH-linked assay are summarized in Supplementary Fig. S1 and Supplementary Table S1.

A conserved feature of Rubisco catalysis is the required priming (activation) of each catalytic site (E) located at the interface of adjoining paired 50kDa large subunits (L_2_) that arrange as (L_2_)_4_ tetrad cores and are capped at either end with tetrads of 15kDa small (S) subunits to form an ~520kDa L_8_S_8_ complex (Andersson and Backlund, 2008). Activation proceeds via the slow and reversible binding of non-substrate CO_2_ to the ε-amino group of a conserved L-subunit Lys201 producing a carbamate (EC) that is rapidly stabilized by Mg^2+^ to produce a tertiary complex (ECM) capable of RuBP binding and enolization and its subsequent carboxylation or oxygenation (Andersson, 2008). *In vivo*, the pool of inactive Rubisco comprises decarbamylated catalytic sites (E) and ECM complexes binding inhibitory sugar-phosphate molecules (ECMI) (Parry *et al.*, 2008) (Fig. 1). Examples of these inhibitors include the catalytic misfire product xylulose 1,5-bisphosphate (XuBP) and the ‘nocturnal’ or ‘shade’ inhibitor 2'-carboxy-d-arabinitol 1-phosphate (CA1P) produced under low light and darkness (Gutteridge *et al.*, 1986; Moore and Seemann, 1992; Pearce, 2006; Andralojc *et al.*, 2012). Binding of RuBP to E also leads to the production of catalytically stalled ER complexes (Laing and Christeller, 1976; Jordan and Chollet, 1983). Release of these sugar-phosphate molecules is catalysed by Rubisco activase (RCA) via ATP hydrolysis (Parry *et al.*, 2008; Mueller-Cajar *et al.*, 2014). Following their RCA-facilitated release, the rebinding of XuBP and CA1P is prevented by the enzymes XuBPase (Bracher *et al.*, 2015) and CA1Pase (Salvucci and Holbrook, 1989; Moore and Seemann, 1992). The enzyme CA1Pase is also able to metabolize the inhibitor pentadiulose-1,5-bisphosphate (PDBP; Andralojc *et al.*, 2012), a relatively labile oxygenation byproduct whose inhibitory relevance *in vivo* remains indeterminate but is a significant contaminant of non-pure RuBP (Kane *et al.*, 1998). Conditions that stimulate Rubisco inactivation include increasing temperature (increased XuBP and PDBP production), low illumination (stimulated CA1P synthesis), and elevated CO_2_ (possibly increases ER levels) (Crafts-Brandner and Salvucci, 2000; Salvucci and Crafts-Brandner, 2004; Kim and Portis, 2005; Parry *et al.*, 2008).

Extrapolating aspects of cellular biochemistry from leaf gas exchange measurements using available C_3_ and C_4_ photosynthesis models is highly reliant on accurately knowing the content and catalytic properties of the carboxylation and decarboxylation enzyme activities (Farquhar *et al.*, 1980; Salvucci and Crafts-Brandner, 2004; Sharkey *et al.*, 2007). Here we appraise and refine the methods for assaying PEPC, PEPCK, and Rubisco activity and activation status using NADH-linked spectrometric assays. We apply these refined assay methods to leaf extracts from C_3_ and C_4_ grasses to demonstrate their applicability in accurately measuring variations in the carboxylation/decarboxylation biochemistries of leaves of differing ontogeny.

## Materials and methods

### Plant seeds and growth conditions

Seeds for *Panicum bisulcatum* and *Megathyrsus maximum* were obtained from the Australian Plant Genetic Resources Information System (QLD, Australia) and Queensland Agricultural Seeds Pty. Ltd (Toowoomba, Australia), while the seeds for tobacco (*Nicotiana tabacum,* cv Petit havana), maize (*Zea mays* cv Kelvedon Glory), and wheat (*Triticum aestivum* cv Y70) were sourced locally. The seeds were sown in 2–5 litre pots of commercial self-fertilizing potting mix at 5–7 d intervals to obtain plants of different ages to sample simultaneously. The plants were grown in a glasshouse at set 28/22 ^o^C day/night temperatures under natural illumination during November and December in Canberra, Australia. Plants were watered regularly, with the addition of Hoaglands nutrients to mature plants every 2 d.

### Leaf harvesting, protein extraction, and protein assay

Samples of known area (0.3 or 0.5cm^2^) were harvested using brass cork borers (Met-App Metalware, Melbourne) from different aged leaves on the same day, 5–7h into the light period. The samples were rapidly frozen in liquid nitrogen before storing at –80 °C. For assays of Rubisco activity and content as well as the activity of PEPC and NADP-ME, the soluble leaf protein was extracted using ice-cold 2ml glass homogenizers (Wheaton) into 0.5–1ml of ice cold, N_2_-sparged extraction buffer [50mM EPPS-NaOH, pH 8.0, 0.5mM EDTA, 2mM DTT, 1% (v/v) plant protease inhibitor cocktail (Sigma-Aldrich), and 1% (w/v) polyvinylpolypyrrolidone (PVPP)] containing 0, 2, 5, or 10mM MgCl_2_. The lysate was rapidly centrifuged for 0.5, 2, or 5min (16 000 *g*, 4 °C), and 10 µl of the soluble leaf protein was assayed for initial and total Rubisco activities (see below) and 50 µl to measure Rubisco content by [^14^C]CABP (2-C-carboxyarabinitol 1,5-bisphosphate) binding as described (Whitney *et al.*, 2001). Protein content was measured against BSA standards using a Coomassie dye binding assay (Pierce). The leaf area extracted in 1ml of buffer was generally 0.3cm^2^ (*T. aestivum*), 0.5cm^2^ (*N. tabacum*), 0.6cm^2^ (*P. bisulcatum*), and 0.9cm^2^ (*P. bisulcatum*, *M. maximus*, and *Z. mays*)

### Phosphoenolpyruvate carboxylase assay

Maximal PEPC activities were measured using an NADH-coupled assay as previously described (Ashton *et al.*, 1990). Extraction was performed using the same extraction buffer as described for Rubisco containing 5mM MgCl_2_, and samples were incubated at room temperature or on ice for 0, 5, 10, and 20min before adding 10 µl of extract to initiate assays.

### NADP-malic enzyme assay

Maximal NADP-ME activity was determined in a coupled NADP assay as previously described (Ashton *et al.*, 1990; Pengelly *et al.*, 2012). Briefly, NADP-ME activity in leaf extracts prepared as described above was assayed in 50mM Tricine-KOH pH 8.3, 5mM malic acid, initiating with 10mM MgCl_2_.

### Phosphoenolpyruvate carboxykinase assay

**Fig. 2.**
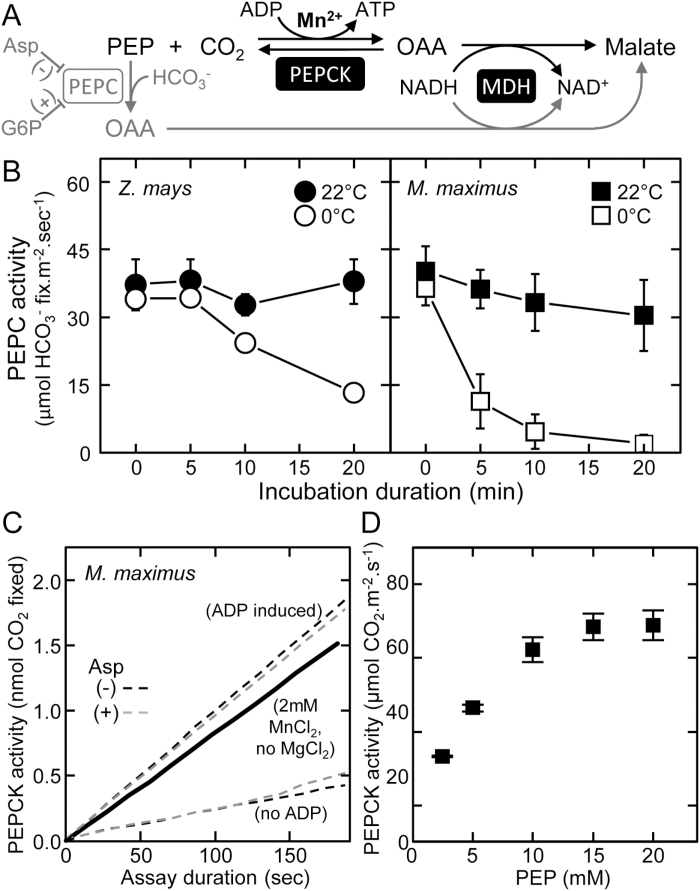
Optimizing the measurement of PEPC and PEPCK activities in leaf extract. (A) Summarizing the commonality of the malate dehydrogenase- (MDH) coupled, NADH oxidation-linked assay to quantify the carboxylation of phosphoenolpyruvate (PEP) into oxaloacetate (OAA) by PEP carboxykinase (PEPCK; in black) and PEP carboxylase (PEPC; in gray), an enzyme inhibited (–) by aspartate (Asp; Huber and Edwards, 1975) and activated (+) by glucose-6-phosphate (G6P). (B) Effect of storage temperature (room temperature, 22 °C, or ice, 0 °C) and time on PEPC activity in the soluble protein from young leaves from mature *Z. mays* and *M. maximus* plants (*n*=3 biological replicates ±SD; see leaves m3b and c3b in Fig. 5A for examples). (C) Representative assay of PEP carboxylation by PEPCK measured in leaf soluble protein extract using the no MgCl_2_ method of Sharwood *et al.* (2014) ( black line) and the modified ADP method of this study in assays with (gray dashed lines) or without (black dashed lines) 5mM aspartate (a PEPC inhibitor, A). (D) Response of PEPCK activity to [PEP] in *M. maximus* leaf soluble protein. To prevent PEPC interference ensure: 1) No MgCl_2_ in assay and extraction buffers, 2) MnCl_2_ up to 5mM in extraction and assay buffers, 3) pH of extraction and assay buffers <7.0, 4) No glucose-6-phosphate, and 5) include aspartate in assay buffer.

The maximal activity of PEPCK was measured in the carboxylase direction using a method adapted from Chen *et al.* (2002) and Walker *et al.* (2002) as described by Sharwood *et al.* (2014) in an NADH-coupled assay as depicted in Fig. 2A. To remove interference with PEPC, MgCl_2_ was excluded from the extraction and assay buffers. PEPC background activity was determined by assaying for PEPC activity as above with and without MgCl_2_, with no PEPC activity observed when MgCl_2_ is omitted from the extraction and assay buffer (data not shown). For PEPCK assay, leaf discs were extracted in 50mM HEPES pH 7.0, 5mM DTT, 1% (w/v) PVPP, 2mM EDTA, 2mM MnCl_2_, and 0.05% Triton. PEPCK activity from leaf extracts was measured in assay buffer [50mM HEPES, pH 7.0, 4% mercaptoethanol (w/v), 100mM KCl, 90mM NaHCO_3_,1mM ADP, 2mM MnCl_2_, 0.14mM NADH, and malate dehydrogenase (MDH; 6U; 3.7 μl)] after the addition of 15mM PEP. An optimal PEP concentration was determined in separate assays titrated with 2.5–20mM PEP.

### Rubisco activity assays

Rubisco activity was measured at 25 °C using an NADH-coupled enzyme assay (Kubien *et al.*, 2011; Supplementary Fig. S1 at *JXB* online) with the rate of NADH oxidation monitored at 340nm using a diode array spectrophotometer (Agilent model 8453). The RuBP used in the assays was either that synthesized and purified as previously described (Kane *et al.*, 1998) or commercially supplied (Sigma; R0878). Assays were performed in 1ml cuvettes containing 0.48ml of assay buffer [100mM EPPS-NaOH, pH 8.0, 10mM MgCl_2_, 0.2mM NADH, 20mM NaHCO_3_, 1mM ATP, pH 7.0, 5mM phosphocreatine, pH 7.0, and 4% (v/v) coupling enzymes] (see Supplementary Table S1). RuBP (0.4mM) was included in the cuvettes used to measure initial Rubisco activity, with 20 µl of soluble leaf protein sample added to start the assays. To measure total Rubisco activities, 20 µl of leaf protein was first activated for 10–15min in RuBP-free assay buffer before initiating Rubisco activity measurements by adding RuBP to 0.4mM. The Rubisco carboxylation rate was determined using the equation:

$$\text{mol RuBP consumed }\cdot \;{\text{min}}^{-1}\;=\left(\frac{\Delta OD_{340}}{4\left(6.22\times 10^{-3}M^{-1}\right)}\right)$$ (1)

which uses the extinction coefficient of NADH (6.22×10^3^ M^−1^ cm^−1^), the rate of change of absorbance at 340nm per minute (ΔOD_340_), and accounts for the four NADH molecules oxidized per RuBP carboxylated by Rubisco in the coupled assay (Supplementary Fig. S1). Substrate-saturated Rubisco carboxylase activity in the same leaf soluble protein was measured by ^14^CO_2_ fixation assays as described (Sharwood *et al.*, 2008). The carboxylation turnover rate (*k*_cat_^c^) was determined from the Rubisco activity measured by either the NADH-coupled enzyme assay or the ^14^CO_2_ fixation assay divided by the Rubisco active site content in the assay as quantified by [^14^C]CABP binding (Ruuska *et al.*, 1998). Time course measurements of Rubisco activity over 30min at 25 °C were undertaken to confirm the functional integrity of Rubisco in the leaf protein extracts.

### Rubisco activase purification and assay

Tobacco RCA was expressed and purified from *Escherichia coli* as described (Baker *et al.*, 2005). Two-stage assays similar to that described by Barta *et al.* (2011) were used to assess if sugar-phosphate Rubisco inhibition in tobacco leaf protein extracts influenced measurements of total Rubisco activity. In the first assay stage, 50 μl of leaf extract was incubated in a 0.5ml final volume with 80 µg ml^–1^ RCA (or BSA in the RCA-free controls) for 2min at 25 °C in ATPase assay buffer [100mM EPPS, pH 8.0, 20mM KCl, 5mM MgCl_2_, 6% (w/v) polyethylene glycol (PEG) (mol. wt 3350Da)], 2mM PEP, 0.2mM NADH, 2mM ATP, 1% (v/v) of a pyruvate kinase/lactate dehydrogenase mixture (PK, 745U ml^−1^; LDH, 906U ml^−1^, Sigma-Aldrich). In the second assay stage, 100 μl of the RCA leaf or BSA leaf protein reactions were added to the NADH-linked Rubisco assays and the total activities compared. Control assays examining tobacco RCA activation of purified tobacco Rubisco ER complexes are described in Supplementary Fig. S2.

### Statistical analysis

Statistical analysis was carried out using one- or two-way ANOVA (Statistica, StatSoft Inc. OK, USA). Means were grouped using a post-hoc Tukey test.

## Results and Discussion

### Optimizing the assay of PEPC and PEPCK activities in total soluble leaf protein

Measuring PEPC and PEPCK activities separately in soluble leaf protein extracts using NADH-linked spectrophotometric assays is complicated by their common requirement for substrate PEP (Fig. 2A) particularly in assays containing Mg^2+^ and Mn^2+^ at physiological concentrations (Muhaidat and McKown, 2013). Determining maximal PEPCK activity in soluble leaf protein extracts is typically achieved by assaying the decarboxylation reaction of PEPCK, requiring purified protein free of other C_4_ enzymes such as PEPC (Ashton *et al.*, 1990; Chen *et al.*, 2002). In contrast, measures of PEPC rates free of PEPCK activity can easily be made by omission of ADP from the assay (Fig. 2A). As *in vitro* measures of PEPC are sensitive to low temperature storage (Hatch and Oliver, 1978), we examined the PEPC activity in soluble leaf protein from *Z. mays* and *M. maximus* stored either at room temperature (22 °C) or on ice (0 °C) (Fig. 2B). When incubated at 22 °C for 20min, there was little or no loss of PEPC activity evident in replica leaf samples from either *M. maximus* or *Z. mays.* In contrast, storage of the leaf protein extracts on ice significantly reduced PEPC activities (measured at 25 °C), particularly in *M. maximus* where >65% of PEPC activity was lost after 5min at 0 °C (Fig. 2B). As a result of its sensitivity to low temperature, all assays of PEPC activity were performed on rapidly extracted (homogenized in <0.5min and centrifuged for 0.5min at 4 ^o^C) leaf soluble protein without storage on ice.

In C_4_ plants with phosphoenolpyruvate carboxykinase (PCK) physiology (e.g. *M. maximus*), PEPCK is the dominant decarboxylase enzyme that utilizes ATP hydrolysis during its reversible decarboxylation of OAA (Fig. 2A). In contrast to the alkali pH 8 preference of PEPC (Greenway *et al.*, 1978), the activity of PEPCK is optimal at pH 7 and 80% lower at pH 8.0 (Ray and Black, 1976; Pierre *et al.*, 2004). To minimize, possibly preclude, PEPC activity, the extraction and measurement of PEPCK carboxylase activity was undertaken at pH 7.0 with Mg^2+^ (required for PEPC activity) omitted and replaced with 2mM Mn^2+^, a PEPCK cofactor (Fig. 2A). Under these conditions, stable rates of PEPCK were obtained in assays initiated by the addition of PEP (solid line, Fig. 2C) (Sharwood *et al.*, 2014).

Alternative PEPCK analyses were undertaken where the assays were initiated with ADP, not PEP (Fig. 2C, black dashed line). Omission of ADP in control assays produced background rates of apparent PEPC activity (Fig. 2C, lower black dashed line); however, the addition of 5mM aspartate (Fig. 2C, grey dashed line) or 5mM glucose-6-phosphate (and MgCl_2_) which inhibit and stimulate PEPC activity, respectively (Fig. 2A), had a negligible effect on the measured activities. This suggests that the background ‘no ADP’ PEP carboxylase activities observed arise from PEPCK activity that is utilizing residual ADP in the soluble protein extract. Consequently, we propose that extracting leaf protein in pH 7.0 buffer with no MgCl_2_ and assaying in an MnCl_2_-containing buffer at the same pH is sufficient to measure PEPCK activity with little or no contaminating PEPC activity.

Assays containing 2.5–25mM PEP were used to determine that a saturating concentration of 15mM PEP was required for maximal PEPCK activity in soluble leaf protein from 1.3mm^2^ of *M. maximus* leaf tissue (Fig. 2D). This saturating PEP concentration is 7-fold higher than the *K*_m_ for PEP measured for *M. maximus* PEPCK (Chen *et al.*, 2002) and was the concentration used in all subsequent PEPCK assays.

### Measuring Rubisco carbamylation status

The carboxylase-limiting component of the C_3_ photosynthesis models stemming from those derived by Farquhar *et al.* (1980) are typically used to derive estimates of *V*_c,max_ [in units of µmol CO_2_ fixed m^−^^2^ s^−1^, that equate to the product of Rubisco sites (µmol CO_2_ fixed m^2^) and *k*_cat_^c^ (s^−1^)]. This measure is extrapolated from the response of the CO_2_ assimilation rate (*A*) with increasing CO_2_ measured by leaf gas exchange, and relies heavily on the temperature response measurements of *k*_cat_^c^, *K*_c_, the *K*_m_ for O_2_ (*K*_o_), and CO_2_/O_2_ specificity (S_c/o_) made for tobacco Rubisco (Sharkey *et al.*, 2007). The universal suitability of these parameters is now in question given the substantial variation observed in the temperature response of these parameters among plant Rubiscos (Walker *et al.*, 2013; Boyd *et al.*, 2015; Perdomo *et al.*, 2015). Moreover, differences in *V*_c,max_ are primarily attributed to variations in Rubisco content and generally overlook differences in the activation status of Rubisco *in vivo*, despite its critical influence on estimates of *V*_c,max_ and *in vivo* determined *k*_cat_^c^ (Bernacchi *et al.*, 2001; Salvucci and Crafts-Brandner, 2004).

As indicated in Fig. 1, within an illuminated leaf chloroplast the catalytic sites of Rubisco (shown as E) are primarily CO_2_–Mg^2+^ activated (ECM) and capable of RuBP catalysis. Binding of inhibitory XuBP or, in darkened leaves, CA1P to ECM produces catalytically inactive ECMI complexes whose activation involves RCA-catalysed dissociation of the sugar-phosphate ligands which are then degraded by substrate-specific enzymes (Jordan and Chollet, 1983; Gutteridge *et al.*, 1986; Edmondson *et al.*, 1990; Bracher *et al.*, 2015). Thus the most abundant form of Rubisco inhibition in illuminated chloroplasts is probably the binding of RuBP to non-carbamylated Rubisco (ER) that renders the catalytic site inactive (Jordan and Chollet, 1983).

The NADH-linked spectrophotometric and ^14^CO_2_ fixation *in vitro* assays typically used to measure Rubisco activation status involve three stages (Fig. 1). The first stage involves the rapid measure of Rubisco activity in rapidly extracted soluble leaf protein. This measures the ‘initial’ Rubisco activity. Saturating amounts of RuBP are included to prevent ECM formation from the ER complexes and ensure that the assays are not RuBP limited (Laing and Christeller, 1976). Replica samples of the leaf protein extracts are then allowed to activate fully by incubating in buffer lacking RuBP but containing saturating CO_2_ and Mg^2+^. During the second stage, the inconsequential RuBP levels in the extract enable its dissociation from the ER complexes to allow ECM formation. The third stage measures the ‘total’ Rubisco activity rate. In samples from darkened or stressed leaves, the formation of ECMI complexes can cause significant underestimation of the ‘total’ activities (Parry *et al.*, 1997, 2002; Carmo-Silva *et al.*, 2010) a consideration we sought to avoid in this study by sampling healthy, naturally illuminated glasshouse-grown plant material 6–7h into the photoperiod.

### Optimizing leaf protein extraction for measuring Rubisco activation status

**Fig. 3.**
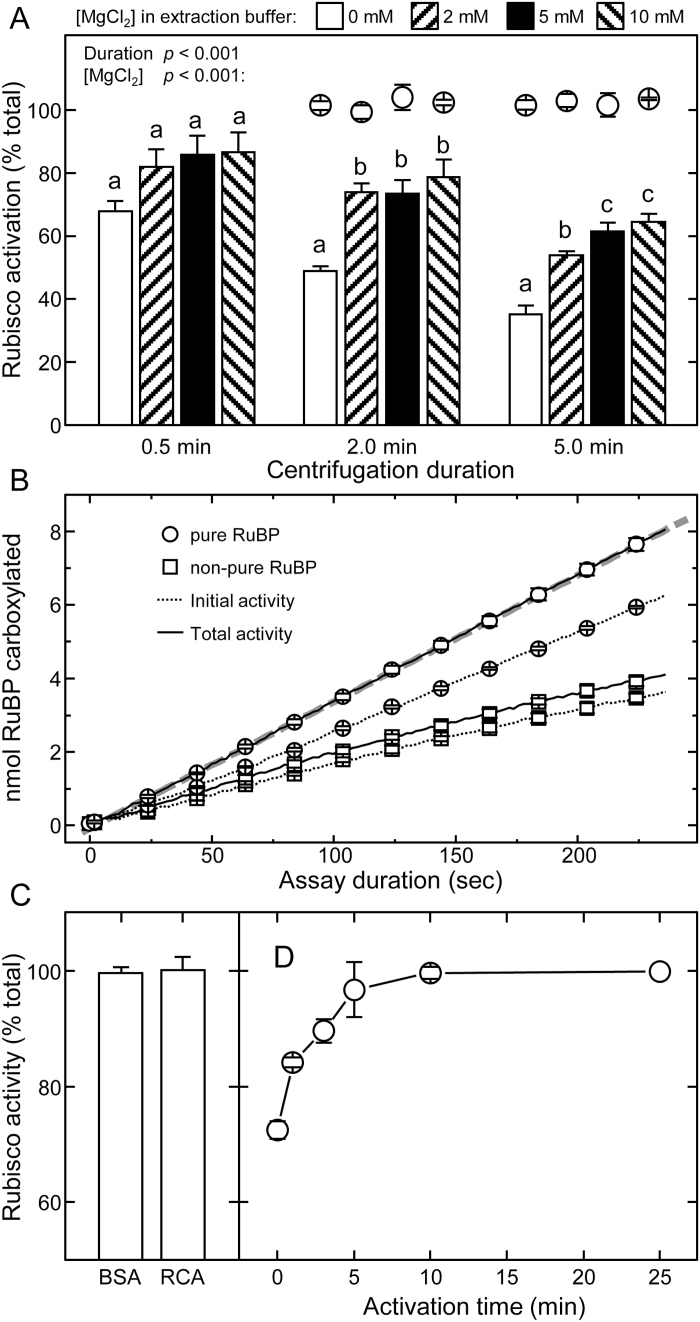
Evaluating the experimental methodology for measuring leaf Rubisco activation status. (A) Appraising how MgCl_2_ inclusion and quickness of soluble leaf protein extraction influences Rubisco activation quantification. NADH-linked assays were performed on N_2_-frozen replica (*n*=5) tobacco leaf discs (0.5cm^2^) taken from a young, nearly fully expanded upper canopy leaf (15cm in diameter) and stored at –80 °C for up to 3 months without effect on recoverable activity. Circles indicate the total activities measured after 10min activation relative to the 0.5min centrifuged sample (B) Representative NADH-linked spectrophotometric measures of initial (dashed lines) and total (solid lines) Rubisco activities made using low purity commercial RuBP (squares) or that purified according to Kane *et al.* (1998) (circles). Rates correspond to protein from 0.9mm^2^ of leaf with a Rubisco active site concentration in each assay of ~34.4nM (i.e. a *k*_cat_ of 2.2s^−1^). (C) Incubation of leaf protein extract for 2min at 25 °C with 80 μg ml^–1^ of either BSA or purified tobacco Rubisco activase (RCA) had no effect on the measured rates of fully activated Rubisco, while the same treatment re-activated >80% of inhibited ER complexes formed using a comparable concentration of purified tobacco Rubisco (Supplementary Fig. S2). (D) Change in the activity status of Rubisco in tobacco soluble protein activated at 25 °C for up to 25min. Data in (C) and (D) are the averages (±SE) from analyses with three separate leaf samples expressed as a percentage of the total activities measured after 10min (C) and 25min (D) activation. For (A), the significance level (*P*) for the [MgCl_2_] and centrifugation duration factors are shown. Letters indicate the ranking (lowest=a) of means within each centrifugation duration using a post-hoc Tukey test. Values followed by the same letter are not significantly different at the 5% level (*P*>0.05).

Analyses undertaken using tobacco leaves highlighted the requirement for speed and inclusion of ~5mM Mg^2+^ during leaf protein extraction for accurate measurements of initial Rubisco activity (Fig. 3A). As shown in Fig. 1, formation of ECM is initiated by the slow and reversible carbamylation of Lys201 in the catalytic site of E followed by the rapid binding of Mg^2+^. To avoid Rubisco carbamylation occurring during extraction, the leaf soluble protein is extracted in N_2_-sparged (i.e. CO_2_-free) buffer. It was hypothesized that the exclusion of MgCl_2_ during extraction would undesirably produce EC complexes from which the activating CO_2_ would dissociate to form inactive E. Indeed, the omission of MgCl_2_ in the CO_2_-free extraction buffer led to significantly lower measurements of initial Rubisco activity (70% of maximum activity) after just 0.5min relative to those extracted with 2–10mM MgCl_2_ (82–85% of maximum activity; Fig. 3A). Extending the extraction (centrifugation) period to 5min reduced all initial Rubisco activity measurements, more so in those extracted without MgCl_2_ (35% of maximum activity) compared with those extracted with MgCl_2_ (55–60% of maximum activity). In all treatments, very similar total activity rates were attained, indicating that the varying extraction conditions did not compromise Rubisco integrity (Fig. 3A, circles). These findings caution against the omission of MgCl_2_ when assaying for initial activities. Furthermore, potential inaccuracies are incurred with extended centrifugation times of >0.5–1min following extraction, emphasizing the need for rapid leaf protein extraction and assay.

### The importance of using purified RuBP

Using pure RuBP devoid of inhibitory impurities such as PDBP is critical for accurately measuring Rubisco catalysis *in vitro* (Kane *et al.*, 1998; Andralojc *et al.*, 2012). As observed by Scales *et al.* (2014), by using pure RuBP the measured rates of initial and total activity remain relatively linear over a 4min assay period (Fig. 3B). This (i) indicates that insignificant levels of the catalytic misfire products XuBP or PDBP are produced over the assay period; (ii) indicates that Rubisco activity in the assay is stable; and (iii) confirms the observations of Laing and Christeller (1976) that inclusion of saturating RuBP levels (0.2–0.4mM) in the ‘initial’ assays prevents Rubisco activation. In contrast, Rubisco activities measured in the same extracts using a commercial source of non-pure RuBP (Fig. 3B, squares) declined rapidly, presumably due to PDBP contamination (Andralojc *et al.*, 2012). The use of non-pure RuBP further compromised the quantification of *k*_cat_^c^ (2.2s^−1^ with pure RuBP versus 1.3s^−1^ with non-pure RuBP) and the calculated activation status of Rubisco (75±2% with pure RuBP versus 83±3% with non-pure RuBP). Use of non-pure RuBP should therefore be avoided in order to avoid underestimating the carbamylation status (Rowland-Bamford *et al.*, 1991; Anwaruzzaman *et al.*, 1996; Ruuska *et al.*, 2004; Sulpice *et al.*, 2007) and activity (Rintamäki *et al.*, 1988; Uemura *et al.*, 2000) of Rubisco.

### Insignificant levels of ECMI complexes accumulate in illuminated, non-stressed leaves

The *k*_cat_^c^ value of 2.2s^−1^ was reproducibly quantified for tobacco Rubisco using the NADH-coupled assay. This is ~30% lower than those typically measured by ^14^CO_2_ fixation assays (Sharwood *et al.*, 2008; Whitney *et al.*, 2011). Incubation of the leaf protein extracts with purified tobacco RCA or BSA (control) showed no difference in the measured total Rubisco activities (Fig. 3C). In corresponding control assays, the RCA treatment was able to reactivate ER inhibited Rubisco fully over 10min (Supplementary Fig. S2). This indicates that the lower *k*_cat_^c^ was not due to residual ECMI complexes in the leaf protein extract. To confirm this, the same tobacco soluble leaf protein was used to quantify *k*_cat_^c^ by the ^14^CO_2_ assay method of Sharwood *et al.* (2008). As indicated in Table 1, the expected *k*_cat_^c^ of 3.1s^−1^ for tobacco Rubisco was obtained by the ^14^CO_2_ assay. This finding questions the accuracy of the NADH-coupled assay for quantifying Rubisco carboxylase activity, a deficiency also evident in the comparative measurements made by Lilley and Walker (1974). Indeed, published *k*_cat_^c^ values determined by the NADH-coupled assay for cyanobacteria (Emlyn-Jones *et al.*, 2006) and plant (Pearce and Andrews, 2003) Rubisco are also 20–25% lower than those measured by ^14^CO_2_ fixation (Whitney *et al.*, 1999; Mueller-Cajar and Whitney, 2008). To ensure that the differences were not due to components in the leaf extracts interfering with the coupling enzymes, comparative assays were undertaken in triplicate (technical repeats) using tobacco Rubisco purified by ion exchange chromatography (see the legend to Supplementary Fig. S2). Again, the *k*_cat_^c^ values determined by the NADH-coupled assay (1.9±0.2s^−1^) were 30% lower than that quantified by ^14^CO_2_ fixation assays (2.7±0.1s^−1^). This suggests that substrate limitations for one or more of the enzymes in the NADH-linked assay limit its potential for accurately quantifying *k*_cat_^c^, possibly the rate of 3-PGA reduction (Lilley and Walker, 1974).

**Table 1.** Comparative values of Rubisco *k*_cat_^c^ at 25 °C quantified by the NADH-linked and ^14^CO_2_ fixation assays

Plant species	Photosynthetic biochemistry	*k*_cat_^c^ (±SE s^−1^)				Significance (*P*)
		NADH-linked assay		^14^CO_2_ assay		
Tobacco	C_3_	2.15±0.02 b	*n*=24	3.07±0.06 b	*n*=23	<0.001
*P. bisulcatum*	C_3_	1.79±0.03 a	*n*=22	2.72±0.11 a	*n*=7	<0.001
*M. maximus*	C_4_-PCK	3.85±0.09 e	*n*=9	5.17±0.17 d	*n*=10	<0.001
*T. aestivum*	C_3_	2.70±0.06 c	*n*=9	3.59±0.04 c	*n*=6	<0.001
*Z. mays*	C_4_-NADP ME	3.70±0.10 d	*n*=9	5.46±0.10 d	*n*=6	<0.001

Values (means ±SE) obtained using NADH-linked and ^14^CO_2_ assays were compared by one-way ANOVA, and the significance level (*P*) is shown.

Species’ means obtained by each of the assay types were ranked separately using a post-hoc Tukey test. Values followed by the same letter are not significantly different at the 5% level (*P*>0.05).

*n*=number of leaf protein samples (biological replicates) analyzed.

### The need for measuring Rubisco activation rate and stability over time

**Fig. 4.**
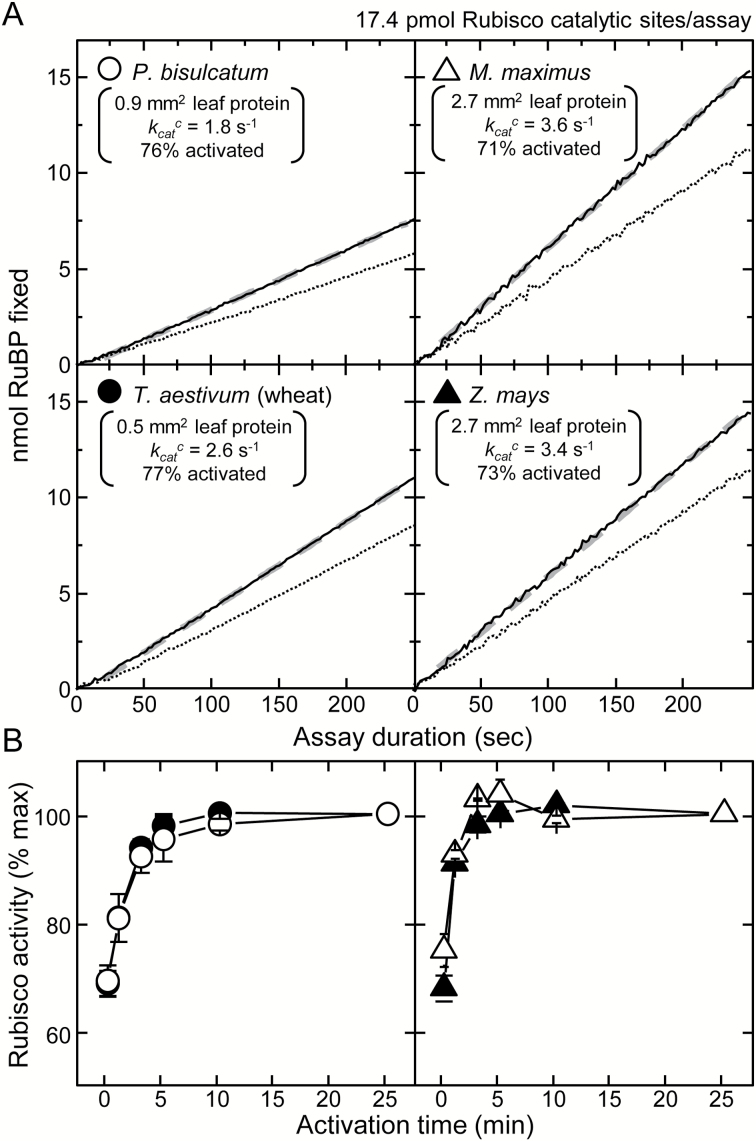
Variations in Rubisco content and activation status during leaf ontogeny can account for variations in the *in vivo* estimates of *V*_*c*_^max^. (A) Representative NADH-linked assay data showing the linear RuBP carboxylation rates in assays of initial (dashed lines) and total (solid lines, after 10–15min activation) Rubsico activities at 25 °C using soluble leaf protein from both C_3_ (*P. bisulcatum*, *T. aestivum*) and C_4_ (*M. maximus*, *Z. mays*) monocotyledon species. Shown are details of the calculated Rubisco activation status (% of maximum), the derived carboxylation rates (*k*_cat_^c^, quantified from the slope of the fitted linear regression, gray dashed line, divided by Rubisco content quantified by [^14^C]CABP binding) and the area of leaf protein required to attain the 17.4 pmol Rubsico catalytic sites used to normalize the plotted data to highlight the variations in *k*_cat_^c^ between each species (see also Table 1). (B) Response of Rubisco activation and activity in the soluble leaf protein of each species following incubation at 25 °C. Shown is the average (±SE) of analyses from three separate leaf samples for each species expressed as a percentage of the maximum activities measured after 25min activation.

Given that the NADH-coupled assay allows for the continued ‘real-time’ monitoring of both initial and total Rubisco activity (Fig. 3B), it was used to examine the activation rate and stability of Rubisco activity at 25 °C in the soluble protein of leaves from the C_3_ species *P. bisulcatum* and *T. aestivum* (wheat), and the C_4_ species *M. maximus* and *Z. mays.* Linear rates of initial and total Rubisco activities were reproducibly found for each sample (Fig. 4A), with the rates of NADH oxidation significantly lower in the C_4_ samples due to their low Rubisco contents. As shown in Fig. 4A, the *k*_cat_^c^ for both C_4_ Rubiscos were seen to be higher than those of their C_3_ counterparts when the activities were corrected for Rubisco content (quantified by [^14^C]CABP binding).

Like tobacco, full activation of Rubisco in the soluble protein extracted from wheat and *P. bisulcatum* required 10min incubation at 25 ºC (Fig. 4B, circles). In contrast, full activation of Rubisco in the *M. maximus* and *Z. mays* leaf protein required only 3–5min (Fig. 4B). Whether the faster rate of Rubisco activation in both C_4_ species arises from a lower RuBP binding affinity remains a subject for future investigation. Nevertheless, variation in the time needed to activate Rubisco fully in leaf protein extracts questions whether shorter incubation times (e.g. 3min) are sufficient to evaluate Rubisco activation status accurately and extrapolate ECMI levels (Parry *et al.*, 1997, 2002; Carmo-Silva *et al.*, 2010; Galmes *et al.*, 2011; Scales *et al.*, 2014).

### The *k*_cat_^c^ of C_4_ plant Rubisco exceeds that of C_3_ Rubisco

The *k*_cat_^c^ values of Rubisco from the C_4_ species examined in this study were significantly faster relative to that of each C_3_ plant Rubisco (Table 1). Notably the NADH-coupled assay measures of *k*_cat_^c^ were again 30–35% lower than corresponding *k*_cat_^c^ measurements made using ^14^CO_2_ fixation assays (Table 1). Statistical ranking of the catalytic speed indicated that the C_3_ Rubisco from *P. bisulcatum* is slower than that of tobacco, with wheat outperforming both (*P*<0.001). In contrast to its ancestral *P. bisulcatum* Rubisco, the *k*_cat_^c^ for *M. maximus* Rubisco is ~2-fold higher but similar to the *k*_cat_^c^ of maize Rubisco despite originating from different biochemical subtypes and evolutionary origins (Table 1).

### How do PEPC, Rubisco content, and activation status vary with leaf age?

**Fig. 5.**
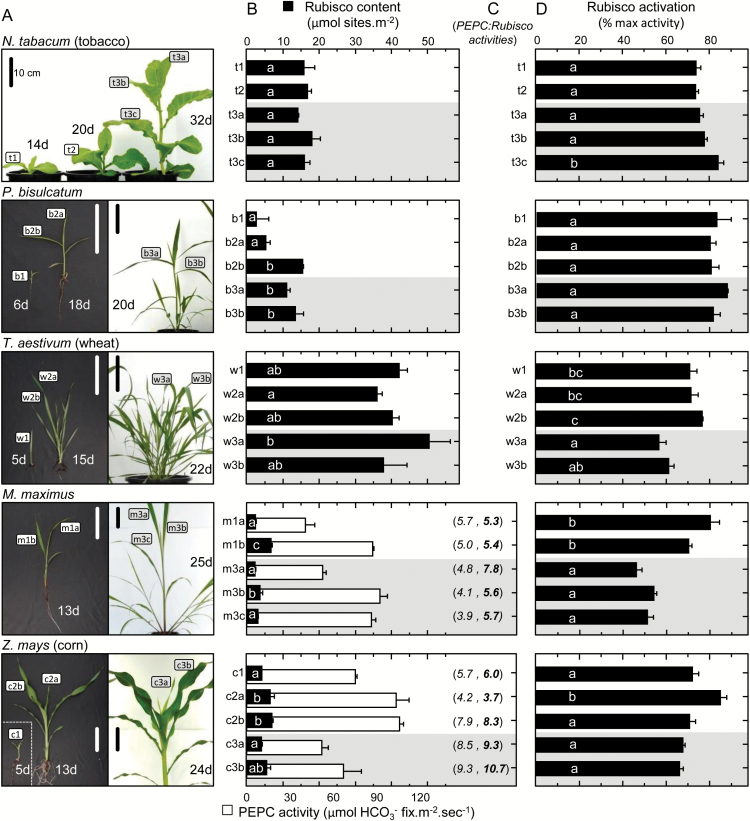
Variation in PEPC activity, Rubisco content, and Rubisco activation status with leaf ontogeny and development in C_3_ and C_4_ plants. (A) Pictures showing the leaves at differing stages of ontogeny and plant development (as labeled) that were analyzed for (B) Rubisco content (black bars, determined by [^14^C]CABP binding) and PEPC activity (white bars). (C) For both C_4_ species, the corresponding PEPC:Rubisco activity ratios are shown in parenthess; the first value is determined from the rates measured using the NADH-linked assays (in italics) and the second value (in bold) takes into account the higher Rubisco *k*_cat_^c^ values quantified using ^14^CO_2_ assays (Table 1) and (D) the activation status of Rubisco in each leaf analyzed. The age of each plant (days, d) post-cotyledon emergence is indicated with the scale bar=10cm. All data are averages (±SD) of *n*=3 leaf discs taken from each leaf (or for the juvenile samples b1, c1, and w1, from replica plantlets). Regions shaded gray in (B) and (D) indicates data for leaves sampled from more mature plants. For (B) and (D), letters indicate the ranking (lowest=a) of means within each species using a post-hoc Tukey test. Values followed by the same letter are not significantly different at the 5% level (*P*>0.05). The levels of Rubisco measured correlate with those previously measured in the leaves of tobacco (Whitney *et al.*, 2011), *P. bisulcatum* (Pinto *et al.*, 2014), *M. maximus* (Pinto *et al.*, 2016), and maize (Sharwood *et al.*, 2014).

Rubisco comprises a significant but variable N investment in plant leaves. In tobacco, wheat, and rice, Rubisco comprises 20–30% of the leaf N, which is equivalent to 30–60% of the leaf soluble protein (Evans, 1989; Makino *et al.*, 2003; Whitney *et al.*, 2011), while in C_4_ plants it is 5–10% (Ghannoum *et al.*, 2005). The lower Rubisco requirement of C_4_ plants stems from their CCM that enables them to operate under near saturating CO_2_ concentration, which has facilitated the evolution of increased Rubisco *k*_cat_^c^ (Ghannoum *et al.*, 2005). Consistent with these findings, the variation in Rubisco content with leaf ontogeny and at different locations in the canopy of *M. maximus* and maize (Fig. 5A) was 3- to 25-fold lower than that measured in C_3_ species (tobacco, *P. bisulcatum*, and wheat, Fig. 5B). Among the C_3_ plants, significantly higher levels of Rubisco were measured in wheat relative to tobacco and *P. bisulcatum*, with the latter grass showing the greatest variation in Rubisco content (per leaf area) in the leaves from juvenile and mature plants.

The variation in Rubisco with leaf ontogeny evident in both C_4_ species was somewhat mirrored by differences in their PEPC activities (Fig. 5B), resulting in similar PEPC:Rubisco activity ratios of ~3.9–5.7 in *M. maximus* that were more varied in maize (4.2–7.9 in juvenile plant leaves and 8.5–9.3 in the young leaves of exponentially growing plants; Fig. 5C). This ratio is typically used as an indication of the CO_2_ supply to the CCM in C_4_ plants and is normally in balance to minimize leakage of fixed CO_2_ (von Caemmerer, 2000; von Caemmerer *et al.*, 2014). In both *M. maximus* and *Z. mays*, the PEPC:Rubisco ratio tended to increase during ontogeny, in particular when the ratio is adjusted with regard to differences in Rubisco activation status (Fig. 5C). The higher Rubisco:PEPC ratio in mature *Z. mays* leaves relative to *M. maximus* may arise from their varying C_4_ biochemistry and/or evolutionary origin, a consideration beyond the objectives of this study.

Despite being produced in lower abundance, the activation status of C_4_ Rubisco was similar or lower than that measured in the C_3_ species. Similarly, low levels of Rubisco activation (~45–55%) have been measured in other C_4_ species (von Caemmerer *et al.*, 2005; Carmo-Silva *et al.*, 2010) that correlate with those measured in mature *M. maximus* and *Z. mays* leaves (Fig. 5D). Higher Rubisco activation levels (~70–80%) were measured in the juvenile C_4_ plant leaves. These levels matched those measured in tobacco and *P. bisulcatum*, where little variation in Rubisco activation status was found among the leaves sampled. In contrast, significant variation in Rubisco activation status was observed among the upper wheat panicle leaves, where Rubisco activation was significantly lower than those sampled from juvenile plants (Fig. 5D).

The level of variation in Rubisco content and activation status with leaf ontogeny identified within this explorative study using plants grown under non-stress conditions emphasizes the importance of determining these parameters to compare meaningfully values of *V*_c,max_ derived by extrapolation from leaf gas exchange (*A*–C_i_ curves) for different biological samples. As demonstrated by Whitney and Sharwood (2014), quantifying the leaf Rubisco content is best achieved using the [^14^C]CABP binding methods as densitometry methods following PAGE separation of Rubisco are highly imprecise, unless appropriately calibrated. Accurate quantification of Rubisco site content in the *in vitro* assays of Rubisco activity are also critical for quantifying *k*_cat_^c^. This parameter also provides a number of quality checks as reduced measures of *k*_cat_^c^ provide a useful indicator of reduced leaf sample viability (as found if ultra-cold temperatures are not maintained during transfer and storage at –80 °C) and incomplete activation (e.g. insufficient activation time and/or presence of significant levels of ECMI complexes in the sample).

### Variation in NADP-ME and PEPCK activities in *Z. mays* and *M. maximus*.

**Fig. 6.**
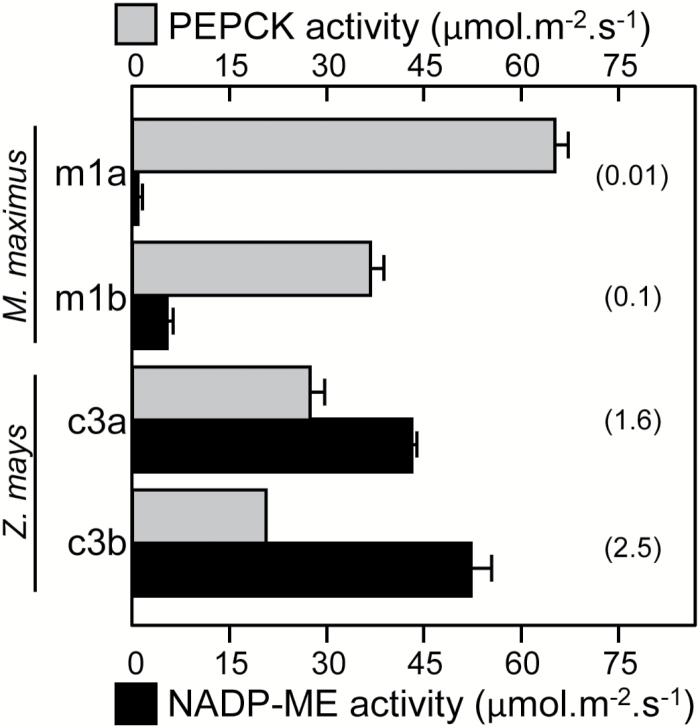
Maximal activity of the decarboxylases in C_4_ grasses. Comparison of PEPCK (gray) and NADP-ME (black) activities in differing aged *Z. mays* and *M. maximus* leaves (*n*=3, ± SE). The leaves analyzed are shown in Fig. 4A. The PEPCK:NADP-ME ratio activities are indicated in parentheses.

Different aged leaves sampled from mature *M. maximus* and *Z. mays* plants were analysed for maximal NADP-ME and PEPCK activities (Fig. 6). Higher PEPCK activities were measured in the younger leaves from both species, with as much as 2-fold higher activity measured in the *Z. mays* samples. Conversely, NADP-ME levels were characteristically >10-fold higher in *Z. mays*, consistent with its C_4_-NADP-ME photosynthetic biochemistry. While the low NADP-ME activity in *M. maximum* probably arose from an anaplerotic reaction, the significance of the PEPCK activity in *Z. mays* is not yet fully understood. Prior analysis of *Z. mays* exposed to salinity stress and shade treatments showed that there is plasticity in PEPCK contents and activity (Sharwood *et al.*, 2014). This suggests that the PEPCK decarboxylation pathway may serve a role in responding to stressful environmental cues (Bellasio and Griffiths, 2014).

### Conclusion

In this study, we demonstrate the need for carefully considering the experimental requirements needed to measure accurately, and reproducibly, the activity of key carboxylase and decarboxylase enzymes that are commonly used to evaluate physiological and biochemical parameters between plant samples. Of particular relevance to C_3_ and C_4_ photosynthetic modelling studies is how Rubisco content and activation can vary significantly with leaf ontogeny, in particular in C_4_ plants where Rubisco activation appears characteristically low. Here we show that full Rubisco activity is recoverable *in vitro* without the need for RCA when extracted with no RuBP. We therefore propose that Rubisco inactivation in the chloroplasts of non-stressed, illuminated leaves is primarily attributable to ER complex formation. Removing Rubisco inhibitors using Na_2_SO_4_ and PEG treatments (Parry *et al.*, 1997, 2002; Carmo-Silva *et al.*, 2010; Galmes *et al.*, 2011; Scales *et al.*, 2014) that can potentially harm recoverable activity might therefore be unnecessary using the *in vitro* assay conditions described in this study.

As summarized in Supplementary Fig. S3, we identified the core requirements for measuring Rubisco activation status (fast extraction, include ~5mM MgCl_2_, use pure RuBP, activate for 10min), PEPC (pH 8, 22 °C post-extraction), PEPCK (pH 7, >2mM Mn^2+^ no Mg^2+^, 15mM PEP), and NADP-ME activities using NADH-linked assays. We highlight how an unresolved limitation in the NADH linked assay underestimates Rubisco *k*_cat_^c^ by >20%. We also emphasize the advantage of quantifying Rubisco by [^14^C]CABP binding to normalize Rubisco activities per active site (i.e. *k*_cat_^c^) as it serves as a quality control indicator of sample integrity and full Rubisco activation. Understandably, the assay and extraction conditions used in this study probably need optimization for other plant samples where additives and conditions (pH, temperature) are required to sustain, or promote, enzyme activities (Supplementary Fig. S3). As shown here by the differing assay requirements of PEPC and PEPCK, this optimization should also assess the compatibility of additives on the activity of each enzyme measured.

## Supplementary data

Supplementary data are available at *JXB* online.

Table S1. Details for the preparation and storage of coupling enzymes used in the NADH-linked spectrophotometric assay of Rubisco activity.

Figure S1. Overview of the NADH-linked enzyme-coupled spectrophotometric assay for measuring Rubisco activity.

Figure S2. Time-dependent activation in vitro of inhibited tobacco Rubisco–RuBP (ER) complexes by RCA.

Figure S3. Core requirements for measuring Rubisco activation status, PEPC, PEPCK, and NADP-ME activities.

Supplementary Data
