# Supplementary material for: Improved analysis of C4 and C3 photosynthesis via refined in vitro assays of their carbon fixation biochemistry
Source: J Exp Bot. 2016 Apr 27;67(10):3137–48. doi: 10.1093/jxb/erw154 (PMC4867899; doi:10.1093/jxb/erw154)
Supplement: Supplementary Data [file supp_67_10_3137__index.html]

Improved analysis of C4 and C3 photosynthesis via refined in vitro assays of their carbon fixation biochemistry — Improved analysis of C4 and C3 photosynthesis via refined in vitro assays of their carbon fixation biochemistry — Supplementary Data 

# Improved analysis of C4 and C3 photosynthesis via refined *in vitro* assays of their carbon fixation biochemistry

## Supplementary Data

Data files

- supplementary\_table\_S1\_figures\_S1\_S3.pdf - Supplementary Data
