# Supplementary material for: Improved analysis of C4 and C3 photosynthesis via refined in vitro assays of their carbon fixation biochemistry
Source: J Exp Bot. 2016 Apr 27;67(10):3137–48. doi: 10.1093/jxb/erw154 (PMC4867899; doi:10.1093/jxb/erw154)
Supplement: Supplementary Data [file supp_erw154_supplementary_table_S1_figures_S1_S3.pdf]

Supplemental Data

Table S1. Details for the preparation and storage of coupling enzymes used in the NADH-linked spectrophotometric assay of Rubisco activity (see Figure S1).

**Step**	Enzyme	Example supplier	Units per assay*	Total units required for 400 assays.	Example activities supplied	Amount required per prep**
**1.**	Centrifuge the following enzymes that are supplied as ammonium sulfate suspensions (16,000g, 4°C, 15 min)					
	3-phosphoglyc-erate kinase (PGK)	Sigma P7634	25	10,000	~3400 Units/ml	~3.0 ml
	Triose-phosphate isomerase/ α-Glycerophosphate dehydrogenase (TIM/G3PDH)	Sigma G1881-5Ku	~55	22,500 (TIM)	~1200 Units/ml TIM	~6.6 ml
			~20	7,750 (G3PDH)	~400 Units/ml G3PDH	
**2.**	Dissolve the pelleted enzymes in 10 ml of CE buffer (50mM Eppes-NaOH pH 7.8, 1mM EDTA, 1mM DTT) and add the following enzymes					
	carbonic anhydrase (CA)	Sigma C3934	0.1 mg	not applicable		40 mg
	creatine phosphokinase (CPK)	Sigma C3755	25	10,000	~360 Units/mg	~30 mg
	glyceraldehyde 3 phosphate dehydrogenase (GAPDH)	Sigma G2267-10KU	25	10,000	~80 Units/mg	~125 mg
**3.**	Dialyze the enzyme mix in 500 ml CE buffer at 4°C for 4 h					
**4.**	Dialyze the enzyme mix in 500 ml CE-glycerol buffer (*i.e.* CE buffer containing 20% (v/v) glycerol) at 4°C for 4 h then recovering the enzyme mix from dialysis (its volume would have reduced) and increase its volume to 8 mL with CE-glycerol buffer before dispensing in 40 x 0.2 ml aliquots, freezing in liquid N_2 and storing at -80°C					

*assumes final assay volume of 1 ml.

** assumes use of 20 µl of coupling enzymes per 1 ml assay and that the final volume of the coupling enzyme preparation is 8.0 ml

Figure S1. Overview of the NADH-linked enzyme coupled spectrophotometric assay for measuring Rubisco activity.

For each RuBP carboxylated by Rubisco, two molecules of 3-phosphoglycerate (PGA) are produced whose conversion to two molecules of glycerol 3-phosphate (glycerol3P) require oxidation of four NADH molecules into NAD^+. The conversion is catalyzed by the coupling enzymes 3-phosphoglycerate kinase (PGK), glyceraldehyde 3-phosphate dehydrogenase (GAPDH), triose phosphate isomerase (TIM) and α-glycerophosphate dehydrogenase (G3PDH). Phosphocreatine (creatine-P) and creatine phosphokinase (CPK) provide an ATP regenerating system. A method for preparing the coupling enzymes and example commercial sources of each enzyme is provided in Table S1. While these enzymes are supplied in excess, the results of this study suggest substrate limitations for one or more of the enzymes limit the potential of the assay to accurately quantify maximal values of Rubisco k_{cat}^{c} (Table 1).

Figure S2. Time dependent activation *in vitro* of inhibited tobacco Rubisco-RuBP (ER) complexes by RCA.

Tobacco Rubisco was rapidly purified by anion exchange chromatography as described (Sharwood *et al.*, 2008) and dialyzed (10,000 MW cutoff) into storage buffer (40 mM Eppes-NaOH, pH8, 1 mM EDTA, 20% (v/v) glycerol) at 4°C for 4hr. The Rubisco concentration was quantified by ^{14}C-CABP binding (Sharwood *et al.*, 2008). The purified Rubisco was diluted to an active site concentration of 10 μM and incubated with 500 μM RuBP for 1 hour at 4°C to form inhibited ER complexes. The ER samples (50 μL) were then diluted 10-fold into assays containing 80 μg/ml RCA or BSA as described in the main text. After incubating 0, 1, 2, 5 or 10 min at 25°C 20 μL of the ER-RCA or ER-BSA reactions were diluted into 0.5 mL NADH-linked Rubisco assays and their Rubisco carboxylase

activities compared (expressed as a % of the maximal Rubisco activity determined for pure Rubisco fully activated with 20 mM $NaHCO_3$ and 20 mM $MgCl_2$ for 30 min at 25°C).

Figure S3. Core requirements for measuring Rubisco activation status, PEPC, PEPCK and NADP-ME activities.

Flow chart describing the key requirements for measuring the key carboxylases and decarboxylases in leaf extracts. Optimization should closely test the compatibility of additives, temperature and pH on the activity of each enzyme measured. For this study we show the optimized extraction conditions and buffers required for measurement of Rubisco activation status, PEPC, PEPCK and NADP-ME activities using NADH linked assays. By quantifying Rubisco by ^{14}C-CABP binding enables normalization of Rubisco activities per active site (*i.e.* k_{cat}^{c}) as it serves as a quality control indicator of sample integrity and full Rubisco activation.

Sharwood R, von Caemmerer S, Maliga P, Whitney S. 2008. The catalytic properties of hybrid Rubisco comprising tobacco small and sunflower large subunits mirror the kinetically equivalent source Rubiscos and can support tobacco growth. *Plant Physiology* **146**, 83-96.

Figure S1

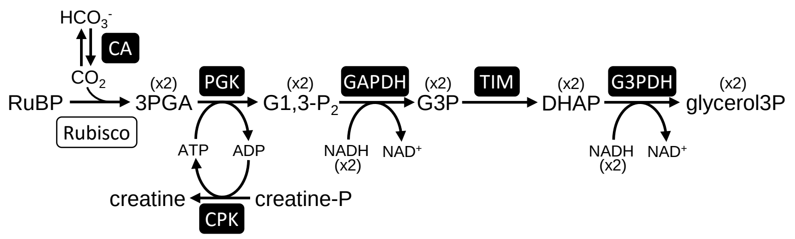
HCO3-
CA
CO2
RuBP
Rubisco
(x2)
3PGA
PGK
(x2)
G1,3-P2
GAPDH
(x2)
G3P
TIM
(x2)
DHAP
G3PDH
(x2)
glycerol3P
ATP
ADP
NADH
(x2)
NAD+
NADH
(x2)
NAD+
creatine
creatine-P
CPK

Figure S2

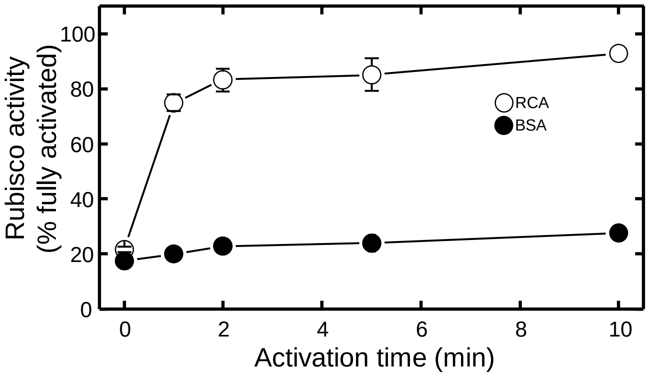

Rubisco activity
(% fully activated)
0
20
40
60
80
100
0
2
4
6
8
10
Activation time (min)
RCA
BSA

Figure S3

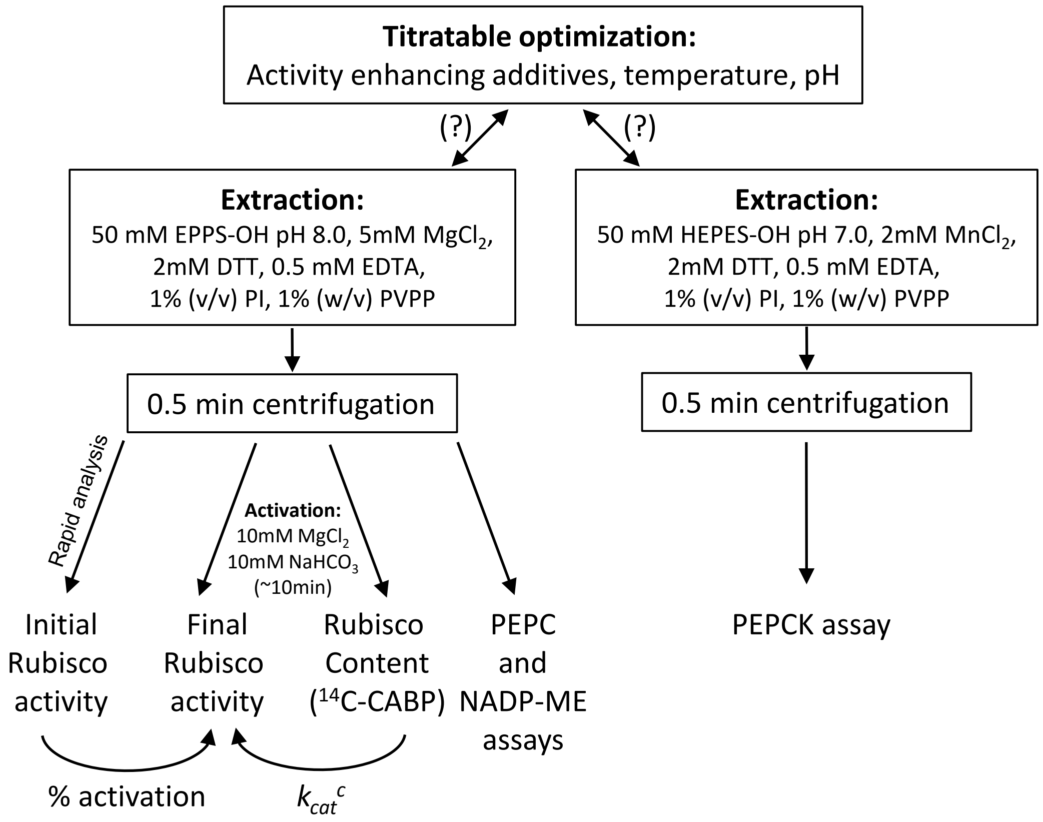

Titratable optimization:
Activity enhancing additives, temperature, pH
(?)
(?)
Extraction:
50 mM EPPS-OH pH 8.0, 5mM $MgCl_2$, 2mM DTT, 0.5 mM EDTA, 1% (v/v) PI, 1% (w/v) PVPP
Extraction:
50 mM HEPES-OH pH 7.0, 2mM $MnCl_2$, 2mM DTT, 0.5 mM EDTA, 1% (v/v) PI, 1% (w/v) PVPP
0.5 min centrifugation
0.5 min centrifugation
Rapid analysis
Activation:
10mM $MgCl_2$
10mM $NaHCO_3$
(~10min)
Initial Rubisco activity
Final Rubisco activity
Rubisco Content (^{14}C-CABP)
PEPC and NADP-ME assays
PEPCK assay
% activation
k_{cat}^{c}
